# Overview of the 2023 Physical Virology Gordon Research Conference—Viruses at Multiple Levels of Complexity

**DOI:** 10.3390/v16060895

**Published:** 2024-06-01

**Authors:** Michael F. Hagan, Roya Zandi, Charlotte Uetrecht

**Affiliations:** 1 Martin Fisher School of Physics, Brandeis University, Waltham, MA 02453, USA; 2 Department of Physics and Astronomy, University of California Riverside, Riverside, CA 92521, USA; 3 CSSB Centre for Structural Systems Biology, Deutsches Elektronen Synchrotron DESY, Leibniz Institute of Virology (LIV), 22607 Hamburg, Germany; 4 Institute of Chemistry and Metabolomics, University Lübeck, 23562 Lübeck, Germany

## 1. Introduction

This review accompanies the Special Issue on the subject of physical virology, which features work presented at the recent Gordon Research Conference (GRC) on this topic. Physical virology is a multidisciplinary approach to understand the fundamental science of viruses and related structures, as well as their practical applications. The Physical Virology GRC brings together researchers in virology, chemistry, biology, computational science, materials science, mathematics, physics, and engineering who (1) share a desire to understand the biophysical mechanisms that regulate and propel virus infections and (2) apply the principles that regulate virus self-assembly and structural dynamics to develop nanotechnology platforms for drug delivery, therapeutics, and vaccines. It was a premier international scientific conference focused on advancing the frontiers of science through the presentation of cutting-edge and unpublished research in talks and poster sessions, prioritizing time for discussion, and fostering informal interactions among scientists of all career stages. The conference was last held in January 2023 in Italy.

The lifecycle of a typical virus is a remarkable manifestation of evolutionary design. In many virus families, the formation of an infectious virus requires that hundreds or thousands of proteins co-assemble with other components, without external guidance or control, into a particular three-dimensional structure [1]. Viral particles must exit a host cell during or after assembly [2], target and infect new cells, and hijack the machinery of infected cells to manufacture the components for new viral particles [3,4,5,6,7]. Some viruses completely redesign the interior of their host cell, creating new compartments and complex processes to transport components [8,9,10,11,12,13,14].

As such, virology requires understanding processes over a wide spectrum of complexity levels. At the global level, viral epidemiology depends on modes of transmission and social networks; at the cellular level, viruses repurpose protein interaction networks and remodel cellular compartments; and at the local level, the assembly and disassembly of a virus are governed by and reveal physical principles. Viruses also bridge multiple scientific disciplines. In biomedicine, understanding viral lifecycles provides essential information for developing antiviral agents that block infection. In more fundamental sciences, viruses are ideal model systems for studying mechanisms underlying self-assembly, genome packaging and release, allostery, membrane dynamics, and the efficient passage of nanoscale particles through membranes. The COVID-19 pandemic has highlighted the need for these cross-disciplinary approaches to understand viral biology [15,16,17], predict their global spread and impact, and develop new treatments. In addition to advancing antiviral strategies and cell biology, the knowledge acquired from these studies and the viral particles themselves are enabling researchers to engineer virus-based platforms for biomedical and nanomaterial applications, such as gene delivery or optoelectronics.

This natural diversity requires the collaborative efforts of researchers with a wide range of expertise. Therefore, this conference brought together scientists and engineers from a spectrum of disciplines bridging basic and applied science. To foster cross-disciplinary communication and identify complementary areas of expertise, sessions included speakers from different disciplines focusing on similar aspects of virology.

In addition to the GRC, a Gordon Research Seminar (GRS) was organized by graduate students for graduate students and post-docs. The GRS was held in conjunction with the GRC and incorporated the goals and diverse scientific background of the parental GRC.

## 2. Overview of the conference

Viruses are ubiquitous, found in every environmental niche compatible with life. Their abundance indicates that viral lifecycles are highly optimized by evolution, capable of adapting to new environments, host immune pressures, or new host organisms while maintaining fundamental physical properties that are crucial for stability and function. Understanding viral lifecycles thus requires research spanning the physical and life sciences, relating the physical principles that govern virus function to biological understanding on atomic to global scales. Similarly, the study of viruses includes applied science and engineering, such as the biomedicine of viral diseases and the reengineering of viruses for synthetic biology or nanomaterial platforms. The COVID-19 pandemic highlighted the critical need for such cross-disciplinary science and engineering approaches for understanding the biology underlying an emergent disease [18], predicting its global spread and impact, and developing novel treatment strategies.

A key goal of the 2023 meeting was to highlight examples of cross-disciplinary approaches focusing on the following three specific aims:

- Promote and broaden interdisciplinary research in physical virology.
- Increase the diversity, equity, and inclusivity (DEI) of the physical virology community.
- Provide training and promote career advancement for early-career researchers.

As pointed out above, the Physical Virology GRC was unique in gathering researchers in virology, chemistry, biology, computational science, materials science, mathematics, physics, and engineering who seek to (1) identifythe biophysical mechanisms that control and drive virus infections and (2) apply the principles that regulate virus self-assembly and structural dynamics to develop nanotechnology platforms for drug delivery, therapeutics, and vaccines. As science becomes more collaborative, we require interdisciplinary interaction focused on the interface between biology and materials science, experimental results with theoretical and computational models, connectivity of in vivo and in vitro results, and single organisms with ecological scales. The 2023 Physical Virology GRC was an ideal event for researchers with very different backgrounds to communicate about a common theme of viruses and related applications within the physical virology community. 

The 2023 meeting (postponed from 2021 due to the pandemic) was the 7th GRC and 6th GRS on Physical Virology. The conference built on its predecessors, which were very positively evaluated by attendees and GRC reviewers. GRC meetings are known for their intimate nature, emphasis on stimulating discussion, and inclusion of participants from different careers and career stages, which was also the case here. The program emphasized discussion (~2/3 of the time dedicated to the presentation and ~1/3 to the respective discussion) for both invited and short talks.

To ensure a well-rounded program with adequate emphasis on both applications and biology, numerous talks were dedicated to applications and cell biology, all while maintaining a clear focus on the fundamental physics dictating viral behaviors. Therefore, the program introduced new topics that directly link fundamental science with applications and bring additional cell biology while maintaining a virology focus. First, in response to the pandemic, several presentations were focused on emerging research and treatment strategies for COVID-19. Second, a new session was dedicated to universal aspects of self-assembly, which covered two sub-topics, i.e., (i) virus-like cages in other organisms, including bacterial microcompartments, and (ii) the de novo design of self-assembling cages. These talks elucidated how the physical principles governing virus assembly apply to other systems, while microcompartments are also relevant to bacterial cell biology. At the same time, microcompartments and human-designed cages have significant practical applications since they can be engineered or reengineered to serve as delivery vehicles or customizable nanoreactors.

Moreover, the concept of “physical virology” was broadened. Over the history of this conference, physical virology has primarily been viewed as the intersection of viral biology and soft matter physics, i.e., the physical principles that govern the self-assembly, mechanics, and structural dynamics of viral particles. However, progress in related fields over the last decade has now led to additional areas that can advance our fundamental understanding of viral lifecycles and biomedical applications of this knowledge. In particular, two sessions were dedicated to expanding the boundaries of physical virology (Table 1). One covered the statistical physics of viral fitness landscapes, how they are reshaped by host immune pressure, and how this knowledge enables rational vaccine design. The other focused on a statistical mechanical understanding of how viruses manipulate the cell protein interaction network during an infection, which is relevant to both a fundamental understanding of viral lifecycles and to identifying novel drug targets.

The increasing diversity of research areas was also in line with increasing DEI by integrating new groups and topics amongst invitees. Diversity was already imprinted at the chair level in terms of gender, region, and scientific area. This helped to ensure a balanced, cohesive program and was similar to the GRS. Moreover, more female scientists (53%—the first time to reach gender parity and higher than at comparable virology conferences [19]), underrepresented groups from the US and internationally, as well as researchers from Asia, Oceania, and South America (16%), participated in the conference. 

The Power Hour was a highlight of the DEI aspect of the conference. While the Power Hour had previously focused exclusively on issues faced by women in science, Prof. Johnna Frierson (Duke) led the 2023 Power Hour to broaden the focus to all underrepresented groups. She then gave an additional, invited presentation to examine how all scientists can work together to address these issues. This included a highly engaging interactive discussion with the audience, which led to the sharing of ideas and perspectives from across the spectrum of social groups, age groups, institution types, and scientific backgrounds. 

Overall, the invited speakers were leading established and early-career international scientists who engage in cross-disciplinary research that spans the complexity levels relevant to viruses, including the physical principles that govern viral assembly, biomechanical properties of virions inside and outside the host, virus–host interaction and co-evolution, and the global ecology and epidemiology of viral infections. The meeting had core components that stressed a fundamental understanding of how viruses function and how to utilize this knowledge for applications such as biomimetics, gene/drug delivery vehicles, and biomedicine. 

The GRS associated with GRC was a success too. The keynote speaker was Barney Graham (NIH, US), whose lecture topics included the intersection between fundamental physical virology and the response to COVID-19. The topics covered a broad spectrum, spanning from viral assembly to infection and nanomaterials and therapeutics, including cutting-edge techniques to probe them. 

The success of the meeting series arose from its cross-disciplinary scientific program. The sessions fostered cross-disciplinary communication and identified complementary areas of expertise by including speakers from different disciplines focusing on similar aspects of virology. For example, sessions were based on an aspect of the viral lifecycle (e.g., Session: Viruses and Membraneous Compartments considered entry/exit of viruses to/from host cells and viral remodeling of host cell membranes); an application (e.g., Session: From Viruses and Host Immune Interactions to Better Vaccines related theoretical immunology and vaccine development); or linking disparate fields based on a common physical principle (e.g., Session: Virus Inspired Designs: from Materials to Other Organisms identified universal aspects of self-assembly by considering bacterial and human-engineered virus-like shells). Hence, sessions had speakers from different areas who complement each other. For example, Chakraborty and Walczak are theorists who study how the interplay between the host immune system and viral fitness landscapes can be used to develop vaccines that inhibit the evolution of viral resistance. On the other hand, Abraham investigates the immune evasion of the SARS-CoV-2 spike and other viral glycoproteins, while Palomares develops viral capsids as vaccines and gene vectors. Similarly, in Session: Viruses and Membraneous Compartments, the speakers had broad, complementary expertise, i.e., Johnson (theoretical chemistry and biophysics), Chlanda (structural virology), Barbirz (physical biochemistry), and Greber (cell biology and biotechnology), but all study how macromolecular complexes such as viruses interact with, pass through, or reshape cellular membranes, and how these processes depend on the physical and mechanical properties of membranes and virions. These sessions were therefore of interest to basic scientists studying the physical principles of self-assembly, cell biologists, drug developers, and clinicians. By highlighting the intersections among these sub-fields, a new interdisciplinary topic was spurred. 

Overall, the GRC presentations highlighted the critical biomedical significance of physical virology. Understanding the mechanisms underlying the viral mechanics and dynamics required for infection and how viruses interact with host immune systems is central to the development of new vaccines and innovative strategies to combat infectious diseases.

## Figures and Tables

**Table 1:** Final program (https://www.grc.org/physical-virology-conference/2023/, accessed on 28 January 2023).

Speaker	Affiliation	Title
Session: “From Virus Dynamics and Mechanics to Breaking Symmetry”; Discussion Leader: Roya Zandi (University of California, Riverside, US)		
Juan Perilla	University of Delaware, US	Elucidating the Molecular Mechanisms of Nuclear Import and Capsid Uncoating by Molecular Dynamics Simulations
Wouter Roos	Rijksuniversiteit Groningen, The Netherlands	Self-assembly of Single Viral Particles
Carmen San Martín	Centro Nacional de Biotecnología (CNB-CSIC), Spain	Seeing and Touching Adenovirus: Complementary Approaches for Understanding Assembly and Disassembly of a Complex Virion
Session: “Virus Inspired Designs: From Materials to Other Organisms”; Discussion Leader: Danielle Tullman-Ercek (Northwestern University, US)		
Fasseli Coulibaly	Monash University, Australia	Rewiring of Flavivirus Virions Supports a New Model of Viral Maturation
Seth Fraden	Brandeis University, US	Synthetic Structural Biology: Exploiting Viral Assembly Principles as an Anti-viral Strategy
Yu Heng Lau	The University of Sydney, Australia	Unexpected Capsid Dynamics and Assembly States in Encapsulin Protein Cages
Cheryl Kerfeld	Michigan State University and Lawrence Berkeley National Laboratory, US	Diversity, Structure, Function and Engineering of Primitive Protein-Based Membranes: Bacterial Microcompartment Shells
Li Chen Cheah	Commonwealth Scientific and Industrial Research Organisation (CSIRO), Australia	Virus Capsids as Scaffolds for Synthetic Biology *(selected short talk)*
Rob de Haas	Wageningen University, The Netherlands	Design of Programmable and Addressable DNA–protein Hybrid Nanostructures *(selected short talk)*
Session: “The GRC Power Hour^TM^” organized by Johnna Frierson (Duke University of School of Medicine, US)		
Session: “Biomedical Applications”; Discussion Leader: Reidun Twarock (University of York, UK)		
Sarah Butcher	University of Helsinki, Finland	Maturation of Tick Borne Encephalitis Virus
Priscilla Yang	Stanford University School of Medicine, US	Antivirals Discovery as an Endeavor in Both Basic and Translational Science
Iris Seitz	Aalto University, Finland	DNA Origami Directed Virus Capsid Polymorphism *(selected short talk)*
Johnna Frierson	Duke University of School of Medicine, US	From the Micro to the Macro
Session: “Principles that Govern Virus Assembly and Disassembly”; Discussion Leader: Jodi Hadden-Perilla (University of Delaware, US)		
Carolyn Teschke	University of Connecticut, US	Using a Scaffold to Build a Capsid
Shee-Mei Lok	Duke-NUS Medical School, Singapore	Flavivirus Assembly and Maturation Process
Vikram Jadhao	Indiana University, US	Computational Studies of Assembly Phenomena in Virus-Based Materials
Lisa Selzer	Gilead Sciences, US	Viral Capsids and Matrix Layers as Antiviral Targets
Kush Coshic	University of Illinois at Urbana Champaign, US	The Structure and Physical Properties of a Fully Packaged Bacteriophage Virion *(selected short talk)*
Marta Bally	Umeå University, Sweden	Herpesvirus Diffusion at the Cell Surface: From Cell-surface Mimics to Live Cell Microscopy *(selected short talk)*
Session: “How Viruses Capture and Release Cargo”; Discussion Leader: Paul van der Schoot (Eindhoven University of Technology, The Netherlands)		
Mauricio Comas-Garcia	Autonomous University of San Luis Potosi, Mexico	SARS-CoV-2 Genome Release and Assembly
Tuli Mukhopadhyay	Indiana University Bloomington, US	Viral Protein Interactions to Assemble Alphavirus Spikes
Robijn Bruinsma	University of California, Los Angeles, US	Dynamic Selection of ssRNA Genome Molecules During HIV Assembly
Session: “From Viruses and Host Immune Interactions to Better Vaccines”; Discussion Leader: Tobias Beck (University of Hamburg, Germany)		
Arup Chakraborty	MIT, US	The immune Response to Mutable Viruses
Jonathan Abraham	Harvard Medical School, US	Mechanisms of Immune Evasion by the SARS-CoV-2 Spike Protein
Laura Palomares	Instituto de Biotecnologia. UNAM, Mexico	Engineering AAV Capsids for Vaccine and Gene Therapy Vector Applications
Aleksandra Walczak	Laboratoire de Physique, École Normale Supérieure, CNRS, France	How Personalized is Your Immune Response?
Edgar Hodge	University of Washington, US	Probing Structural Dynamics by Mass Spectrometry Provides New Insights into HIV’s Structural and Antigenic Diversity *(selected short talk)*
Janine-Denise Kopicki	CSSB Centre for Structural Systems Biology, Hamburg/University of Siegen, Germany	From MHC Characterization to Screening Approaches *(selected short talk)*
Session: “Virus–Host Protein Interaction Networks”; Discussion Leader: Kelly Lee (University of Washington, US)		
Alfredo Castello	University of Glasgow, UK	When Viral RNA Met The Cell: A Story of Protein-RNA Interactions
Pietro Scaturro	Leibniz Institute of Virology, Germany	Orthogonal Proteomics to Decipher the Arbovirus-Infected Landscape
Artur Biela	Jagiellonian University, Poland	Controlling Size and Polymoprhism of a Protein Cage *(selected short talk)*
Carolina Perez Segura	University of Delaware, US	Mechanistic Insights into the CpAM-induced Disruption of the HBV Capsid Revealed by All-atom Molecular Dynamics Simulations *(selected short talk)*
Emmanuelle Quemin	Institute for Integrative Biology of the Cell (I2BC), CNRS UMR9198, France	Assembly of Vaccinia Virus and Dynamics of Structural Rearrangements During Host Cell Entry *(selected short talk)*
Session: “Viruses and Membraneous Compartments”; Discussion Leader: Nicole Tischler (Fundación Ciencia & Vida—Universidad San Sebastián, Chile)		
Margaret Johnson	Johns Hopkins University, US	Quantifying Membrane Bending Along HIV Lattice Assembly Paths
Petr Chlanda	Heidelberg University Hospital, Germany	Minimal structural Components of the SARS-CoV-2 Pore and Assembling Virions
Stefanie Barbirz	MSB Medical School Berlin, Germany	Infection Initiation of Tailed Bacteriophages at Outer Membranes of Gram-negative Bacteria
Urs Greber	University of Zurich, Switzerland	Cell and Virion Mechanics in Virus Infection
Matthew Shin	UC San Diego, US	Pluronic F127 ‘Nanoarmor’ for Stabilization of Cowpea Mosaic Virus Immunotherapy *(selected short talk)*
Edward Partlow	Brandeis University, US	Dynamic Regulation of Influenza A Virion Assembly in Response to Antibody Pressure *(selected short talk)*
Keynote Session: “Connecting Fundamental and Applied Physical Virology”; Discussion Leader: Pedro De Pablo (Universidad Autonoma de Madrid, Spain)		
Stephen Harrison	Harvard Medical School, US	Molecular Mechanisms of Cell Entry by Non-enveloped Viruses
Juliana Carten	Cornell University, US	Interactions Between the SARS-CoV-1 Fusion Peptide and Calcium Can Inhibit Host Membrane Insertion *(selected short talk)*
Chantal Abergel	UMR7256 CNRS-AMU, France	dsDNA Genome Organization in Giant Viruses Infecting Acanthamoeba
